# Interaction of the hydrogen sulfide system with the oxytocin system in the injured mouse heart

**DOI:** 10.1186/s40635-018-0207-0

**Published:** 2018-10-19

**Authors:** Tamara Merz, Britta Lukaschewski, Daniela Wigger, Aileen Rupprecht, Martin Wepler, Michael Gröger, Clair Hartmann, Matthew Whiteman, Csaba Szabo, Rui Wang, Christiane Waller, Peter Radermacher, Oscar McCook

**Affiliations:** 1 Institute of Anesthesiological Pathophysiology and Process Engineering, University Medical School, Helmholtzstrasse 8-1, 89081 Ulm, Germany; 2 Clinic for Psychsomatic Medicine and Psychotherapy, University Medical Center, Ulm, Germany; 3 Department of Anesthesiology, University Medical Center, Ulm, Germany; 4 University of Exeter Medical School, St. Luke’s Campus, Exeter, England, UK; 5 Chair of Pharmacology, Department of Oncology, Microbiology and Immunology, Faculty of Science and Medicine, University of Fribourg, Fribourg, Switzerland; 6 Department of Anesthesiology, University of Texas Medical Branch, Galveston, TX USA; 7 Department of Biology, Laurentian University, Sudbury, ON Canada; 8 Department of Psychosomatic Medicine and Psychotherapy, Paracelsus Medical University, Nuremberg General Hospital, Nuremberg, Germany

**Keywords:** Cystathionine-γ-lyase, GYY4137, Arginine-vasopressin receptor, Vascular endothelial growth factor, Blood glucose, Cardiovascular system

## Abstract

**Background:**

Both the hydrogen sulfide/cystathionine-γ-lyase (H_2_S/CSE) and oxytocin/oxytocin receptor (OT/OTR) systems have been reported to be cardioprotective. H_2_S can stimulate OT release, thereby affecting blood volume and pressure regulation. Systemic hyper-inflammation after blunt chest trauma is enhanced in cigarette smoke (CS)-exposed CSE^−/−^ mice compared to wildtype (WT). CS increases myometrial OTR expression, but to this point, no data are available on the effects CS exposure on the cardiac OT/OTR system. Since a contusion of the thorax (Txt) can cause myocardial injury, the aim of this post hoc study was to investigate the effects of CSE^−/−^ and exogenous administration of GYY4137 (a slow release H_2_S releasing compound) on OTR expression in the heart, after acute on chronic disease, of CS exposed mice undergoing Txt.

**Methods:**

This study is a post hoc analysis of material obtained in wild type (WT) homozygous CSE^−/−^ mice after 2-3 weeks of CS exposure and subsequent anesthesia, blast wave-induced TxT, and surgical instrumentation for mechanical ventilation (MV) and hemodynamic monitoring. CSE^−/−^ animals received a 50 μg/g GYY4137-bolus after TxT. After 4h of MV, animals were exsanguinated and organs were harvested. The heart was cut transversally, formalin-fixed, and paraffin-embedded. Immunohistochemistry for OTR, arginine-vasopressin-receptor (AVPR), and vascular endothelial growth factor (VEGF) was performed with naïve animals as native controls.

**Results:**

CSE^−/−^ was associated with hypertension and lower blood glucose levels, partially and significantly restored by GYY4137 treatment, respectively. Myocardial OTR expression was reduced upon injury, and this was aggravated in CSE^−/−^. Exogenous H_2_S administration restored myocardial protein expression to WT levels.

**Conclusions:**

This study suggests that cardiac CSE regulates cardiac OTR expression, and this effect might play a role in the regulation of cardiovascular function.

## Background

Hydrogen sulfide (H_2_S) is an important regulator of the cardiovascular system and has been shown to be protective in myocardial ischemia-reperfusion injury (I/R) [1–3] and heart failure [4]. In the central nervous system, H_2_S has recently been implicated in the release of both oxytocin (OT) and arginine-vasopressin (AVP), thereby affecting blood volume regulation [5].

In mice, the genetic deletion of cystathionine-γ-lyase (CSE; CSE^−/−^) leads to hypertension [6, 7], and OT knock-out mice (OT^−/−^) are characterized by lower baseline but higher stress-induced blood pressure than wildtype (WT) animals [8]. The heart is known to express CSE [9, 10], and both OT as well as the OT receptor (OTR) [11]. The oxytocin system has protective effects in myocardial I/R injury [12–15], and its downregulation is implicated in dilated cardiomyopathy [16], and hypertension [17], suggesting that reduced levels of OTR may aggravate these pathologies [18].

Pre-traumatic cigarette smoke (CS) exposure has been reported to aggravate organ dysfunction after trauma and hemorrhage [19]. However, equivocal data regarding the regulation of CSE in CS-exposed rodents are available: both its up- and downregulation have been reported [20–23]. Furthermore, detrimental effects of CSE inhibition as well as a benefit from the exogenous administration of H_2_S have been shown [22–24]. Finally, in a model of acute on chronic disease, we recently showed that post-traumatic systemic hyper-inflammation and acute lung injury (ALI) were aggravated in CSE^−/−^ with pre-traumatic CS exposure when compared to wildtype (WT) littermates [7].

Scarce data are only available on the role of the OT system during acute and/or chronic alterations of gas exchange: OT signaling is protective in fetal hypoxemia [25] and hypercapnia-induced tachycardia and hypertension [26], and CS exposure increases myometrial OTR expression [27, 28]. However, no data are available on any of these effects on the cardiac OT/OTR system. Txt not only causes ALI but is also frequently associated with myocardial injury [29, 30]. Therefore, we chose to investigate OTR expression in heart tissue from the most severely affected groups from the aforementioned previous study [7] that included a modulation of the H_2_S system.

## Methods

This is a post hoc study of material available from previous experiments [7] that were performed in adherence to the National Institutes of Health Guidelines on the Use of Laboratory Animals and the European Union “Directive 2010/63 EU on the protection of animals used for scientific purposes.” and authorized by the federal authorities for animal research of the Regierungspräsidium Tübingen (approved animal experimentation number: 1130), Baden-Württemberg, Germany, and the Animal Care Committee of the University of Ulm, Baden-Württemberg, Germany. The experiments were conducted on C57BL/6J mice that were received from Charles River laboratories Germany (Sulzbach, Germany) and homozygous (CSE^−/−^) mutant mice (C57BL/6J.129SvEv) bred in-house [6]. Animals were kept under standardized conditions and were equally distributed in terms of age, body weight, and sex (10–25 weeks, 26 +/− 3 g, male and female). Native animals were anesthetized with sevoflurane (2.5%; Sevorane, Abbott, Wiesbaden, HE, Germany) and buprenorphine (1.5 mg/g; Temgesic, Reckitt Benckiser, Slough, UK), mid-line laparotomy was performed, and animals were sacrificed via venous exsanguination. Hearts were harvested and fixed in formalin for further analysis.

### Cigarette smoke inhalation procedure

All animals underwent CS exposure for 5 days per week over a period of 3 to 4 weeks using a standardized protocol, as described previously [31]. Prior to the blast wave procedure, mice were allowed to recover for 1 week to avoid acute stress effects induced by the CS procedure per se.

### General anesthesia, blast wave, and surgery

All animals received a Txt and were grouped according to wild type (WT) and CSE^−/−^ with CS exposure. Prior to chest trauma WT and knock-out mice (*n* = 8 per group) were anesthetized with sevoflurane (2.5%; Sevorane, Abbott, Wiesbaden, HE, Germany) and buprenorphine (1.5 mg/g; Temgesic, Reckitt Benckiser, Slough, UK), as described previously [31]. Blunt chest trauma was induced by a single blast wave positioned on the middle of the thorax, as described previously [32]. Briefly, a Mylar polyester film (Du Pont de Nemur, Bad Homburg, Germany) was rapidly ruptured by compressed air, thereby releasing a single blast wave to the murine mid-sternal chest to reproducibly induce a lung contusion without serious organ damage. Immediately afterwards, CSE^−/−^ mice received an administration of GYY4137 or an equivalent volume of saline as a single intravenous injection of 50 μg/g [33, 34], and all mice received ketamine (120 mg/g; Ketanest-S, Pfizer, New York City, NY), midazolam (1.25 mg/g; Midazolam-ratiopharm, Ratiopharm, Ulm, BW, Germany), and fentanyl (0.25 mg/g; Fentanyl-hameln, Hameln Pharma Plus GmbH, Hameln, NI, Germany), and were placed on a procedure bench incorporating a closed-loop-system for body temperature control [7, 32, 35]. Lung-protective mechanical ventilation using a small animal ventilator (FlexiVent, Scireq, MO, Canada) was performed via a tracheostomy, as described previously [7, 31, 35]. Surgical instrumentation comprised catheters in the jugular vein, the carotid artery, and the bladder [31]. General anesthesia was titrated to guarantee complete tolerance against noxious stimuli and was sustained by continuous intravenous administration of ketamine, midazolam, and fentanyl to reach deep sedation, fluid resuscitation comprised hydroxyethyl starch 6% (Tetraspan, Braun Medical, Melsungen, HE, Germany) [31]. At the end of the experiment, the animals were exsanguinated and organs were harvested. The heart was cut transversally and was fixed in formalin for immunohistochemistry (IHC).

Hemodynamic and metabolic parameters were recorded hourly, blood gas tensions, acid-base status, glycemia, and lactatemia were assessed at the end of the 4 h period of mechanical ventilation [31]. The clinical data provided for the experimental groups are obtained from the mouse ICU, which requires surgical instrumentation and thus cannot be provided for the native animals.

### Immunohistochemistry

IHC was performed as described previously [32, 36, 37]. After formalin fixation, hearts were dehydrated, embedded in paraffin, and 3 μm sections were cut. Slides were deparaffinized and rehydrated, followed by heat-induced antigen retrieval by microwaving in 10 mM citrate (pH 6). After blocking with 10% goat serum (20 min), OTR, Arginine Vasopressin Receptor 1A (AVPR), and vascular endothelial growth factor (VEGF) expression were analyzed with the following primary antibodies: anti-OTR (rabbit polyclonal, Proteintech, Manchester, UK 1:50), anti-AVPR (rabbit polyclonal, Abcam, Cambridge, UK 1:200), and anti-VEGF (rabbit polyclonal, Abcam, Cambridge, UK 1:200) in diluent (TBS pH = 8, 0.3% Tween 20, 0.1% goat serum). Slide sections containing native and experimental tissue were analyzed concurrently, as well as positive and negative controls. AVPR was analyzed because it shares a 57% homology to OTR and thus, OT can work through AVPR as well [38]. The expression of vascular endothelial growth factor (VEGF) was determined as a mediator of cardiac function [39, 40] and H_2_S is reported to be cardioprotective via a VEGF-dependent pathway [4]. Primary antibodies were detected by a secondary anti-rabbit IgG antibody conjugated to Alkaline Phosphatase; Jackson, ImmunoResearch, West Grove, Pa, USA) and visualized with a red chromogen (Dako REAL Detection System Chromogen Red, Agilent Santa Clara, CA, USA). Counterstaining was performed with Mayers hematoxylin (Sigma, Taufkirchen, Germany). Slides were analyzed using the Zeiss Axio Imager A1 microscope (Zeiss, Jena, TH, Germany). Two distinct 800,000 μm^2^ regions were quantified for intensity of signal by using the Axio Vision 4.8 software. Results are presented as densitometric sum red [31, 32, 36].

### Statistical analysis

Unless stated otherwise, all data are presented as median (quartiles). After exclusion of normal distribution using the Kolmogorov–Smirnov test, intergroup differences were analyzed using the Kruskal–Wallis ANOVA on ranks and, if appropriate, subsequently the Dunn post hoc test for two-tailed multiple comparisons. The significance level was set to *P* < 0.05. Quantitative graphical presentations and statistical analyses were done with GraphPad Prism 5 (GraphPad Software Inc., La Jolla, CA, USA).

## Results

### Physiological data

All injured animals used in this study underwent pre-traumatic CS exposure and Txt. Physiological data are shown in Table 1. CSE^−/−^ mice showed higher heart rates than the WT mice, and GYY4137 did not affect this parameter. CSE^−/−^ mice also had significantly higher MAP than the WT animals, and GYY4137 fell in between the two other groups. CSE^−/−^ mice had lower circulating glucose levels than wildtypes; GYY4137 administration restored circulating glucose to normal levels. Further, CSE^−/−^ mice had reduced lactate levels, less negative base excess and higher pH in comparison to WT, GYY4137 did not have any statistically significant effects on these parameters.

**Table 1:** Physiological data of injured animals (CS exposure + Txt)

	WT	CSE^−/−^	CSE^−/−^ GYY4137	*p* value
Heart rate (beats/min)	330 (316; 356)	402 (390; 410)^a^	395 (363; 438)^a^	0.0140
Mean arterial pressure (mmHg)	57 (55; 59)	84 (74; 89)^a^	75 (63; 88)	0.0044
Glucose (mg/dl)	92 (86; 107)	76 (72; 82)^a^	95 (90; 104) ^b^	0.0186
Lactate (mmol/l)	1.1 (1.0; 1.5)	0.7 (0.6; 0.8)^a^	0.9 (0.8; 1.1)	0.0035
Arterial base excess (mmol/l)	− 10.2 (− 11.0; − 8.5)	−5.7 (− 7.0; − 4.9)^a^	− 6.9 (− 9.4; − 5.0)	0.0107
Arterial pH	7.25 (7.25; 7.28)	7.37 (7.33; 7.41)^a^	7.35 (7.29; 7.37)	0.0059
Urine (g)	0.6 (0.4; 0.9)	1.9 (1.7; 2.6)^a^	1.3 (1.2; 1.7)	0.0037

Data given as median (interquartile range)

^a^Significant to wt

^b^Significant to CSE^−/−^

### Protein expression in the heart

**Fig. 1:**
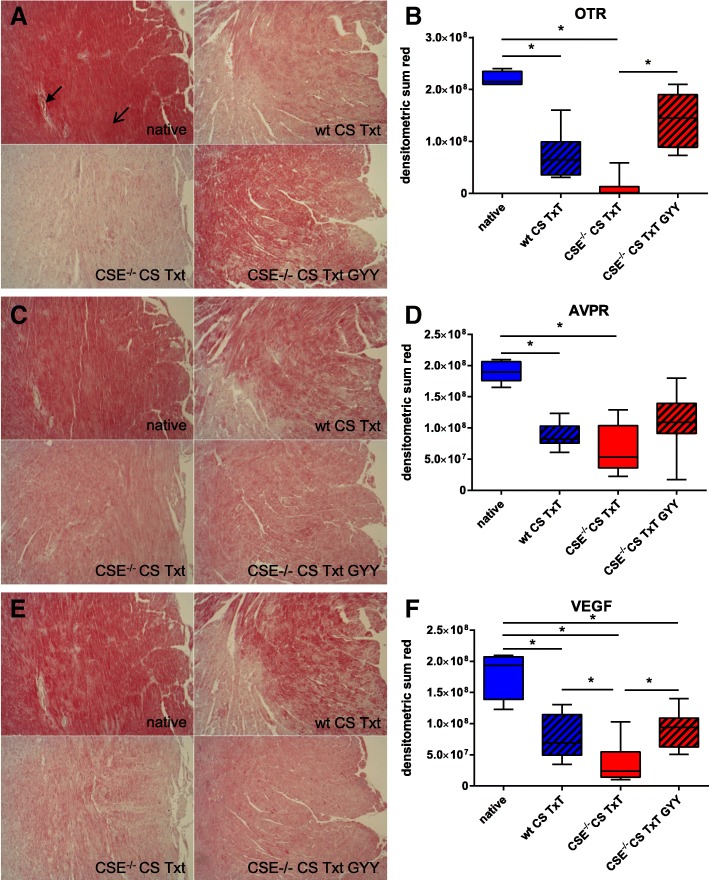
Immunohistochemistry. Exemplary pictures (top left native, top right WT CS Txt, bottom left CSE^−/−^ CS Txt, bottom right CSE^−/−^ CS Txt GYY4137, respectively) of left-ventricular myocardium (ventricular lumen to the right) and densitometric analysis for OTR expression (**a**, **b**), AVPR expression (**c**, **d**), and VEGF expression (**e**, **f**). Data given as box plots (median, interquartile range, minimum and maximum). Boxplots represent *N* = 4 (native) and *N* = 8 (experimental groups). Open arrow cardiomyocyte, bold arrow cardiac microvasculature

Oxytocin receptor (OTR) expression in the heart (see Fig. 1a, b) was constitutive in native animals and could be detected in cardiomyocytes (open arrow) as well as the cardiac microvasculature (bold arrow, Fig. 1a). CS + Txt significantly reduced cardiac OTR expression, and this effect was further enhanced in the CSE^−/−^ animals. Exogenous administration of GYY4137 restored OTR expression so that receptor protein levels did not significantly differ from native animals. Cardiac AVPR expression was also significantly reduced in injured animals, though the effect of CSE deletion was less pronounced GYY4137 administration did not modify this response (see Fig. 1c, d). VEGF expression was reduced upon injury, most pronounced in CSE^−/−^ animals but then restored to WT levels upon GYY4137 treatment (see Fig. 1e, f).

## Discussion

This study was to test the hypothesis if there is a relationship between the H_2_S and the OTR system in the mouse heart in the combined setting of “acute on chronic disease.” The main findings were that Txt after pre-traumatic CS exposure caused (i) a significant downregulation of the cardiac OTR which was (ii) even more pronounced in mice with a genetic CSE deletion of, and that (iii) the administration of the slow H_2_S-releasing compound GYY4137 reversed the effects of CSE deletion.

CSE^−/−^ mice were characterized by higher MAP (75–90 mmHg), which is in accordance with the literature: a genetic CSE deletion leads to hypertension [6], although this effect appears to be context-dependent (anesthesia, handling of the animals etc.) [41]. In C57/BL6 mice undergoing continuous i.v. anesthesia MAP is approx. 60 mmHg [42], which is comparable to the values WT animals in this study. Administration of GYY4137 slightly reduced MAP, preserved or restored the OTR, VEGF, and AVPR in the heart, suggesting cardioprotection as has been reported in myocardial I/R [43–46] and chronic heart failure [47].

Both H_2_S and OT have been implicated in the regulation of energy homeostasis: H_2_S enhances glucose-generating and suppresses glucose-consuming processes leading to increased glucose availability [37]. OT/OTR knock-out mice develop obesity [48], and chronic OT administration led to weight loss in obese monkeys [49]. We and others have shown that hyperglycemia leads to downregulation of CSE expression and reduction of H_2_S formation [37, 50–52]. These results agree with a similar finding for the OT/OTR system: reduced OT levels were reported during hyperglycemia [18, 53].

Equivocal data, however, have been reported on the relationship between H_2_S and the OT system: both the H_2_S liberating salt Na_2_S and the slow-releasing compound GYY4137 inhibited OT effects; however, all the data were obtained in myometrial samples [54–56]. Moreover, You et al. showed an inverse correlation of CSE and OTR expression [57]. In contrast, intracerebroventricular Na_2_S injection not only reduced water intake and stimulated OT release, but also increased plasma levels of AVP and OT [5, 58]. Our findings support these latter results: not only did we observe a more pronounced loss of OTR expression in absence of CSE, but the OTR was restored to native levels through GYY4137 administration.

Due to structural analogy of OT and arginine vasopressin (AVP), the peptides might bind to each other’s receptor [38], and, consequently, we also investigated the AVPR expression. Our results suggest that cardiac AVPR is not as impacted by H_2_S administration as the OTR.

H_2_S has been reported to work through a VEGF-dependent pathway [4] that mediates cardioprotection [39, 40]. VEGF, in the GYY4137 group, as previously mentioned, was restored to WT levels. This suggests a link for the interaction of the H_2_S pathway and the OT/OTR system, in that OT also has been reported to signal through the activation of VEGF [59–61].

## Conclusions

In this preliminary study, performed on post hoc material, we investigated the relationship between CSE, OTR, and H_2_S in the mouse heart after CS exposure and Txt. Genetic CSE deletion led to a pronounced loss of OTR protein expression concomitant with reduced VEGF and AVPR expression. Although the exact mechanisms must be further investigated, our study suggests that cardiac CSE and OTR may interact in cardiovascular (dys)function [10, 18, 37, 49].

## Abbreviations

ALI: Acute lung injury
AVP: Arginine-vasopressin
AVPR: AVP receptor
COPD: Chronic obstructive pulmonary disease
CS: Cigarette smoke
CSE: Cystathionine-γ-lyase
H_2_S: Hydrogen sulfide
IHC: Immunohistochemistry
MAP: Mean arterial pressure
OT: Oxytocin
OTR: OT receptor
Txt: Blunt chest trauma
VEGF: Vascular endothelial growth factor
WT: Wildtype
